# Supplementary material for: A photosensor employing data-driven binning for ultrafast image recognition
Source: Sci Rep. 2022 Aug 24;12:14441. doi: 10.1038/s41598-022-18821-5 (PMC9402579; doi:10.1038/s41598-022-18821-5)
Supplement: Supplementary file 1 — Supplementary Figures. [file 41598_2022_18821_MOESM1_ESM.pdf]

Supplementary Information for

A photosensor employing data-driven binning for ultrafast image recognition

Lukas Mennel[1], Aday J. Molina-Mendoza[1], Matthias Paur[1], Dmitry K. Polyushkin[1], Dohyun Kwak[1], Miriam Giparakis[2], Maximilian Beiser[2], Aaron Maxwell Andrews[2], and Thomas Mueller[1‡]

[1] *Vienna University of Technology, Institute of Photonics, Gußhausstraße 27-29, 1040 Vienna, Austria*

[2] *Vienna University of Technology, Institute of Solid-State Electronics, Gußhausstraße 25, 1040 Vienna, Austria*

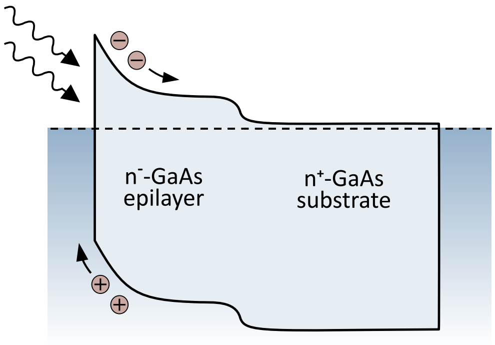

Supplementary Figure S1 | Band diagram of GaAs Schottky photodiode.

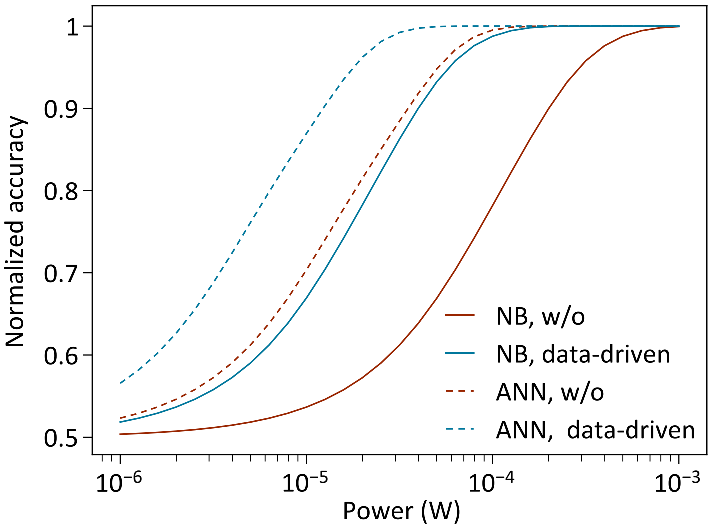

Supplementary Figure S2 | NB versus ANN image sensor. Normalized classification accuracies (MNIST, 14×14 pixels, binary) for the NB classifier (same data as in Figure 5b in the main text; solid lines) and the ANN classifier (dashed lines).
